# Half a Million of Money in Sight

**Published:** 1887-04-23

**Authors:** Rutherford Alcock


### HALF A MILLION OF MONEY IN SIGHT!

By Sir Rutherford Alcock, K.C.B.

The Chairman of the Westminster Hospital has just issued a circular to the various Hospital Committees, in which he states:—"I have been requested by the house committee of this hospital, to forward to you a copy of the following resolution, passed on the 29th ult., at a meeting specially convened for its consideration—'That a letter be sent to the chairmen of the general hospitals of the metropolis dependent on voluntary contributions for their maintenance, inviting them to take into consideration the best means of bringing under the notice of the Charity Commissioners the needs of the said hospitals, with a view to the application of some of the funds of the obsolete City Parochial Charities towards their support.' "

The object of the committee in passing this resolution, it will be seen, was to invite the active co-operation of the other general hospitals of the metropolis dependent wholly, or in part, on voluntary contributions, in an endeavour to bring under the notice of the Charity Commissioners the great need for more funds than the voluntary subscriptions supply, to enable them to keep open the wards now in occupation for the sick poor of the metropolis, and which otherwise are in danger of being partially closed.

This appeal, it is believed, may with great fitness be made to the Commissioners appointed by the Act of Parliament (46 and 47 Vict., ch. 36), passed in 1883, under the title of "City of London Parochial Charities Act." This being an Act to provide for the better application of the parochial charities of the City of London, and under the powers conferred the said Commissioners have been enabled to schedule and take possession of property and moneys to a very large amount, to be applied to "charitable purposes for the benefit of the poorer classes of this metropolis." The hospitals have, it would appear, an undoubted claim upon such funds.

The Commissioners having moreover a wide discretionary power as to their application for the establishment and maintenance of "Convalescent Hospitals," and generally for the improving by the above or any other means which to the Commissioners may seem good, the physical, social, and moral condition of the poorer inhabitants (Sect. 14), they can scarcely decline to entertain the proposed representation. Upon these grounds, and in consideration that a large portion of such funds and endowments were originally devised to benefit the sick poor in the several parishes and districts, it is deemed just and fitting that some portion at least should at once be applied to the alleviation of the great financial distress of many of the principal hospitals, and to their maintenance in a state of efficiency.

If this view should commend itself to your committee, and to the committee of the other associated hospitals, I am to suggest that steps should be taken, in communication with you and the other chairmen, for an early conference, in order to consider, in the terms of the resolution, the best mode of bringing the needs of the said hospitals under the notice of the Charity Commissioners, either by a representation in a collective form of the several administrative committees through their respective chairmen, or in any other mode that may on consideration be deemed preferable.

